# Concomitant circulation of lentiviruses and *Mycoplasma* spp. in dairy goats: a clinical and multi-sample diagnostic approach

**DOI:** 10.1007/s11259-026-11363-5

**Published:** 2026-06-24

**Authors:** Huber Rizzo, Natália Carrillo Gaeta, Jeferson Silva Carvalho, Felipe Gabriel Carneiro Pessoa, Jerônimo Hugo de Souza, Guilherme José da Costa e Silva, Ana Claudia Campos, Edísio Oliveira de Azevedo, Lilian Gregory, Lúcio Esmeraldo Honório de Melo

**Affiliations:** 1 Department of Veterinary Medicine, Federal Rural University of Pernambuco, Recife, PE Brazil; 2 Department of Internal Medicine, School of Veterinary Medicine and Animal Science, University of São Paulo, São Paulo, SP Brazil; 3 Graduate Program in One Health, Santo Amaro University, São Paulo, SP Brazil; 4 Federal Institute of Education, Science and Technology of Bahia, Governador Mangabeira, BA Brazil; 5 Department of Veterinary Medicine, Federal University of Sergipe, Nossa Senhora da Glória, SE Brazil

**Keywords:** Agar gel immunodiffusion, Arthritis, Contagious agalactia, *Mollicutes*, *Mycoplasma agalactiae*, Polymerase chain reaction

## Abstract

The health of dairy goat herds may be compromised by infections caused by small ruminant lentiviruses (SRLV) and *Mycoplasma agalactiae*, particularly under coinfection conditions. This study investigated a dairy goat herd composed of 38 animals with a history of joint enlargement and reduced milk production in Northeastern Brazil. Clinical, serological, molecular, and phylogenetic analyses were performed using blood, milk, synovial fluid, and ocular swab samples. Clinically, arthritis was observed in 28.9% (11/38) of the animals, keratoconjunctivitis in 28.9% (11/38), and mastitis or agalactia in 19.4% (6/31) of the lactating females. For SRLV diagnosis, positivity rates were 31.6% (12/38) by agar gel immunodiffusion (AGID) and 65.8% (25/38) by indirect ELISA, with moderate agreement between tests (K = 0.387; *P* = 0.002). Indirect ELISA for *Mycoplasma* spp. detected antibodies in 34.2% (13/38) of the animals, while PCR identified *M. agalactiae* in 83.3% (5/6) of synovial fluid samples, 66.7% (2/3) of ocular swabs, and 79.3% (23/29) of milk samples. Concomitant clinical manifestations were observed exclusively in coinfected animals. Phylogenetic analysis based on the 16 S rRNA gene supported the molecular identification of *M. agalactiae*. These findings demonstrate the concomitant circulation of SRLV and *M. agalactiae* in the investigated herd and reinforce the importance of combining clinical evaluation with serological and molecular methods applied to different biological samples for the diagnosis of coinfections in goats.

**Supplementary Information:**

The online version contains supplementary material available at https://doi.org/10.1007/s11259-026-11363-5.

## Introduction

Goat farming plays an important role in the economic and social development of several countries, particularly in regions with adverse environmental conditions, such as Northeast Brazil. In these areas, goat production represents an important source of income for rural communities. However, herd health is frequently compromised by infectious diseases, among which small ruminant lentiviruses (SRLV) are particularly relevant due to their significant economic impact. These infections are characterized by a chronic, progressive course that affects multiple organ systems, including joints, the mammary gland, the lungs, and the central nervous system, often resulting in reduced productivity and premature culling (Azevedo et al. 2017).

In addition to lentiviral infections, mycoplasmas represent another important threat to the health of small ruminants. Among the species affecting goats, *Mycoplasma agalactiae* is the most frequent etiological agent of contagious agalactia and is associated with mastitis, arthritis, and keratoconjunctivitis (Migliore et al. 2021). Other relevant species for goat health include *M. conjunctivae*, which causes ocular infections (Giangaspero et al. 2010)d *mycoides* subsp. *capri*, which is commonly associated with respiratory disease (Wagner et al. 2024) and is also considered part of the contagious agalactia complex (Migliore et al. 2021). Regardless of the bacterial species involved, these infections may spread rapidly within herds, resulting in abrupt reductions in milk and meat production and increased animal mortality (Matos et al. 2019).

The diagnosis of these infections generally follows the recommendations of the World Organisation for Animal Health (WOAH). For lentiviruses, agar gel immunodiffusion (AGID) and enzyme-linked immunosorbent assay (ELISA) are recommended methods for antibody detection (WOAH 2018a). For mycoplasma infections, such as those caused by *M. agalactiae*, ELISA is recommended as an indirect diagnostic method, whereas culture and isolation are considered direct diagnostic approaches (WOAH 2018b). Molecular assays, such as polymerase chain reaction (PCR), are valuable tools for the direct detection of pathogens, particularly during the early stages of infection before seroconversion and in situations where culture and isolation are not feasible.

The possibility of coinfection between lentiviruses and mycoplasmas in small ruminants has become an increasing concern (Damasceno et al. 2020). Concurrent infections may intensify clinical manifestations, complicate diagnosis and treatment, increase economic losses, and compromise herd health management practices. However, intermittent shedding of mycoplasmas in samples such as milk (Jaÿ and Tardy 2019), the delayed seroconversion inherent to SRLV infection (Luján et al. 2025), and differences in analytical sensitivity and specificity among diagnostic methods often require the use of combined approaches to confirm infection.

In this context, further studies on the co-occurrence of lentiviruses and mycoplasmas in small ruminants are needed, particularly in dairy goat herds in low- and middle-income countries such as Brazil. Although the diagnostic methods currently available are well established, the integrated application of serological and molecular assays using different biological samples may contribute to a broader understanding of coinfections involving SRLV and *Mycoplasma* spp., supporting more effective diagnostic and control strategies in dairy goat herds.

Therefore, the present study aimed to characterize the clinical and laboratory findings in a dairy goat herd from the State of Pernambuco, Brazil, in which the animals presented clinical manifestations commonly associated with lentiviral and mycoplasma infections, including joint enlargement and reduced milk production.

## Materials and methods

### Study location and herd investigation

In September 2021, a technical visit was conducted to a dairy goat farm in the municipality of Sanharó, in the Agreste region of the State of Pernambuco, Brazil (08°21′38″ S; 36°33′56″ W), following the producer’s report of joint enlargement and reduced milk production among the animals. The herd had recently been purchased from another breeder in the municipality of Pedra, Pernambuco (08°29′50″ S; 36°56′28″ W), approximately 60 km away, about 15 days before the visit. During the visit, a structured interview was conducted with the owner, and the farm facilities and feeding management were inspected. Individual clinical examinations of the animals were performed, and biological samples were collected for laboratory analyses.

### Herd characterization

The herd consisted of 38 adult dairy goats, including Saanen (*n* = 29), Alpine (*n* = 4), Toggenburg (*n* = 3), and crossbred animals (*n* = 2), comprising 36 females and two breeding males. The animals were managed under a semi-intensive production system in a fenced area of approximately 1,000 m², with sparse native pasture and no shaded areas. At night, the goats were confined in a shelter with a dirt floor and partial roofing. The diet consisted of concentrate feed (corn.

and soybean meal), corn silage, mineral supplementation, and water provided *ad libitum*.

### Clinical examination and sample collection

A complete herd anamnesis and physical examination were performed on all 38 goats, with special attention given to joints and mammary glands through palpation. Milk was also evaluated using the strip cup test with a black background (Pugh et al. 2020).

Arthritis was clinically defined as joint enlargement associated with pain or palpation tenderness, postural changes, locomotor difficulty, or prolonged recumbency. Joint temperature was subjectively assessed by palpation of the articular surface. Mastitis was characterized by abnormalities detected during mammary palpation and/or strip cup examination, including the presence of clots and altered milk secretion. In contrast, agalactia was defined as a marked reduction or absence of milk production.

Subsequently, 5 mL of venous blood was collected from 11 animals presenting joint and/or mammary alterations by jugular venipuncture using vacuum tubes containing ethylenediaminetetraacetic acid (EDTA) for hematological analyses and tubes without anticoagulant for the determination of total plasma protein and fibrinogen. Additionally, venous blood samples without anticoagulant were collected from all animals for serological testing. Blood samples were transported in refrigerated cool boxes and processed within 24 h. Serum was obtained by centrifugation at 1,600 g for 10 min, aliquoted into microtubes, and stored at − 20 °C until analysis.

A total of 29 milk samples, seven synovial fluid samples, and three ocular swab samples were also collected. Milk samples were obtained by manual milking after teat disinfection with alcohol and were placed in sterile 10 mL tubes (NMC 2017). Arthrocentesis was performed with the animals restrained on an elevated platform after local trichotomy and perineural anesthesia using 1 mL of 1% lidocaine hydrochloride applied to the medial and lateral regions above the affected joint. Synovial fluid was aspirated using a 40 × 12 mm needle attached to a sterile 10 mL syringe and transferred to sterile 2 mL microtubes. Ocular secretions were collected using sterile swabs by gently rubbing the region below the corneal surface of the affected eye. All samples were kept refrigerated during.

transport and stored at − 20 °C until processing.

### Hematological and biochemical analyses

Hematological and biochemical analyses were performed using samples from 11 goats clinically diagnosed with arthritis. The following parameters were evaluated: hematocrit, hemoglobin concentration, erythrocyte count, erythrocyte indices (mean corpuscular volume and mean corpuscular hemoglobin concentration), and total leukocyte count using a Neubauer chamber. Total plasma protein concentration and fibrinogen levels were determined by refractometry and heat precipitation at 56 °C, respectively (Jain 1993).

### Serological and molecular diagnosis

Serological testing for small ruminant lentivirus infection was performed using the AGID test, according to the manufacturer’s protocol (Biovetech, Recife, Brazil). Results were read between 48 and 72 h under dark-field illumination. Samples showing precipitation lines between the antigen and test wells were considered positive.

Indirect ELISA was performed using commercial kits according to the manufacturer’s instructions (Eradikit™ SRLV Screening and Genotyping Kits, IN3 Diagnostics, Turin, Italy). Screening plates were coated with multi-epitope recombinant peptides derived from matrix (MA), capsid (CA), and transmembrane (TM) proteins from genotypes A and B.

Serology for *Mycoplasma* spp. was performed using an in-house indirect ELISA based on 96-well plates coated with antigen. Cut-off values were established using negative control sera plus 3 standard deviations (Letesson et al. 1997; Castro et al. 1999).

For molecular detection of *Mycoplasma* spp., conventional PCR was performed using milk, synovial fluid, and ocular swab samples. DNA extraction was performed using a boiling method (Fan et al. 1995). Samples were initially screened for *Mollicutes* using PCR primers described by Van Kuppeveld et al. (1992). Positive samples were subsequently analyzed using species-specific PCR assays for *M. conjunctivae* (Giacometti et al. 1999), M. *agalactiae* (Chávez González et al. 1995)d *mycoides* subsp. *capri* (Monnerat et al. 1999). Positive and negative controls were included in all reactions to ensure assay reliability. Amplified products were visualized by agarose gel electrophoresis at 100 V for 35 min.

Selected amplicons were subjected to Sanger sequencing. The obtained sequences were edited and assembled into contigs using Geneious (Kearse et al. 2012), yielding a consensus sequence of the 16 S rRNA gene for the analyzed sample. For phylogenetic analysis, a dataset was constructed that included the sequences generated in this study and representative sequences from 46 *Mycoplasma* species retrieved from GenBank across different countries, totaling 47 sequences. Accession numbers, species identification, and countries of origin are provided in Supplementary Table 1.

Multiple sequence alignment was performed using the MUSCLE algorithm (Edgar 2004) with default parameters, and the alignment was subsequently inspected and edited in BioEdit v7.2.5 (Hall 1999), with trimming of low-quality terminal regions and removal of incomplete sequences or sequences containing extensive ambiguous sites. The final dataset was analyzed in MEGA XI (Tamura et al. 2021). The best-fitting nucleotide substitution model was selected using the Find Best DNA/Protein Models (ML) function based on the Bayesian Information Criterion (BIC) (Kumar et al. 2018; Tamura et al. 2021). Phylogenetic reconstruction was performed using the Maximum Likelihood (ML) method with branch support evaluated by bootstrap analysis with 500 pseudoreplicates (Felsenstein 1985). The resulting tree was edited in FigTree v1.4.4 (Rambaut 2018), collapsing clades corresponding to the same species to improve visualization.

### Statistical analysis

Data were expressed as absolute and relative frequencies. Agreement between AGID and indirect ELISA results was evaluated using Cohen’s kappa coefficient with the irr package in R software (v4.0.5). Associations between laboratory findings and clinical manifestations (arthritis, conjunctivitis, mastitis, and agalactia) were evaluated using Pearson’s chi-square test or Fisher’s exact test when expected frequencies were < 5. All statistical analyses were performed using R software (R Core Team 2021) within the RStudio environment (v.2025.09.2).

## Results

### General clinical findings

A herd composed of 38 goats was evaluated following reports of joint enlargement and a marked reduction in milk production. The herd consisted predominantly of females (94.7%), most of which were of the Saanen breed (76.3%). Among these animals, 47.4% (18/38) presented clinical manifestations compatible with arthritis, keratoconjunctivitis, agalactia, and/or mastitis.

**Fig. 1:**
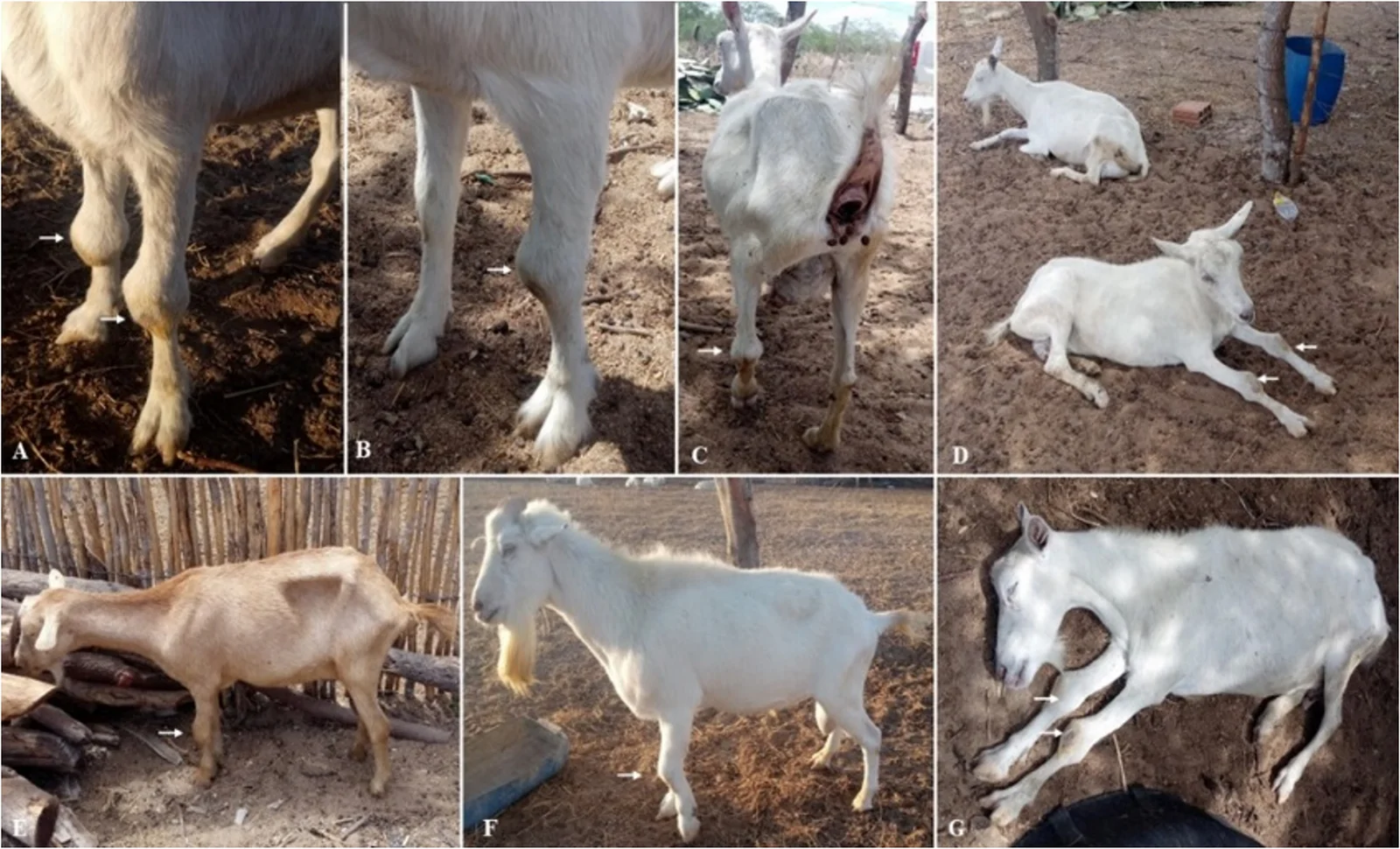
Enlargement of carpal (**A**, **B**, **D**, **E**, **F**, and **G**) and tarsal (**C**) joints observed during clinical examination. Arrows indicate the location of the lesions. Panel **B** shows an edematous carpal joint (white arrow) compared with the healthy contralateral joint. In panel **G**, the goat is shown in prolonged lateral recumbency due to severe joint pain

During physical examination, most animals presented a body condition score between 3 and 4 (71.1%), rectal temperature between 39.0 and 39.9 °C (71.1%), and normal mucous membrane coloration (71.1%). Joint enlargement was observed in 28.9% (*n* = 11) of the goats, mainly affecting both carpal joints (54.5%). The number of affected joints per animal ranged from 1 (45.5%) to 4 (9.1%), with involvement of 2 (27.3%) or 3 joints (18.2%). The affected joints were painful and sensitive to palpation, and some animals presented lameness, reluctance to move, prolonged recumbency, and limb extension due to severe articular involvement. Despite the marked enlargement of the carpal and tarsal joints, marked local hyperthermia was not consistently observed (Fig. 1).

**Fig. 2:**
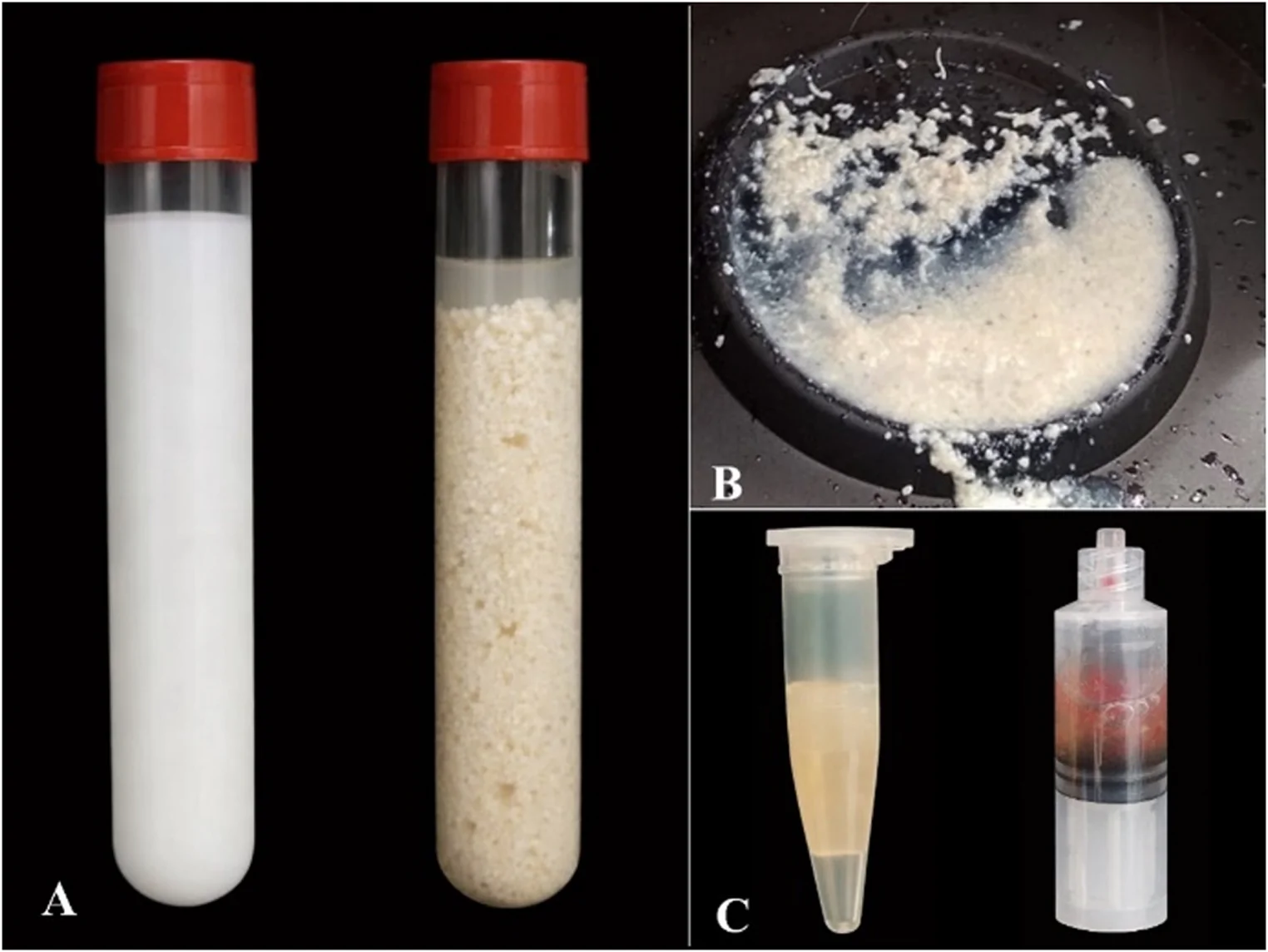
Macroscopic characteristics of clinical samples collected for molecular diagnosis. (**A**) Normal milk (left) and mastitic milk (right) showing phase separation and coagulated/flocculated material. (**B**) Strip cup test demonstrating milk with numerous clots and floccules. (**C**) Synovial fluid samples showing turbid appearance (microcentrifuge tube) and hemorrhagic discoloration (syringe)

**Fig. 3:**
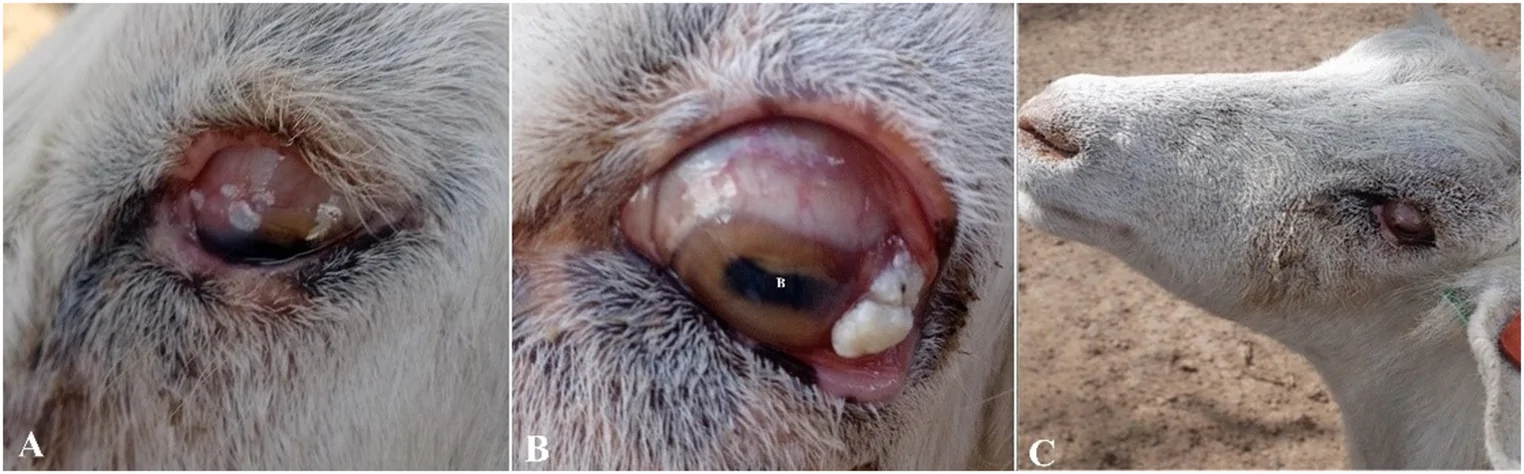
Clinical aspects of keratoconjunctivitis in goats, characterized by conjunctival hyperemia, corneal opacity, episcleral vessel engorgement, ocular discharge, and protrusion of conjunctival tissue

Among the lactating females (*n* = 31), cases of mastitis (9.7%) and agalactia (9.7%) were identified. Milk from goats with mastitis showed a watery appearance, with phase separation and the presence of clots/floccules (Fig. 2). Ophthalmic examination revealed keratoconjunctivitis in 28.9% of the animals, characterized by conjunctival hyperemia, corneal opacity, ocular discharge, and, in some cases, protrusion of conjunctival tissue (Fig. 3). Bilateral involvement was the most frequent presentation (54.5%). In addition, enlargement and softened consistency of superficial lymph nodes, suggestive of caseous lymphadenitis, were observed in 42.1% of the goats, while mammary papilloma’s were identified in 18.4% of the animals (Table 1).

**Table 1:** Distribution of clinical findings in goats (*n* = 38) evaluated in Sanharó, Pernambuco State, Brazil

Variables		Category	% (*N*)
**Sex**	Female		94.7 (36)
	Male		5.3 (2)
**Breed**	Saanen		76.3 (29)
	Alpine (Brown Alpine)		10.5 (4)
	Toggenburg		7.9 (3)
	Mixed breed		5.3 (2)
**Body condition score (BCS)**	1.0		2.6 (1)
	1.5		5.3 (2)
	2.0		13.2 (5)
	2.5		7.9 (3)
	3.0		23.7 (9)
	3.5		23.7 (9)
	4.0		23.7 (9)
**Mucous membranes**	Pale		7.9 (3)
	Normal		71.1 (27)
	Hyperemic		21.1 (8)
**Temperature (°C)**	37.5–37.9		2.6 (1)
	38.0–38.9		21.1 (8)
	39.0–39.9		71.1 (27)
	40.0–40.4		5.3 (2)
**Arthritis**	Affected goats		28.9 (11)
	Left carpus		27.3 (3)
	Right carpus		18.2 (2)
	Bilateral carpi		27.3 (3)
	Left carpus and tarsus		9.1 (1)
	Right carpus and tarsus		9.1 (1)
	Carpi and tarsi		9.1 (1)
**Lactation disorders** (lactating goats, *n* = 31)	Affected goats		19.4 (6)
	Mastitis		50 (3)
	Agalactia		50 (3)
**Keratoconjunctivitis**	Affected goats		28.9 (11)
	Left eye		18.2 (2)
	Right eye		27.3 (3)
	Bilateral		54.5 (6)
**Lymphadenitis**			42.1 (16)
**Papilloma**			18.4 (7)

Based on serological and molecular findings, 33 goats were considered positive for *M. agalactiae* infection. Of these, 13 animals were positive by ELISA, while 29 synovial fluid, ocular swab, and/or milk samples were positive by PCR. In five animals, molecular analysis could not be performed due to the absence of suitable biological samples; two of these animals were seropositive for SRLV and seronegative for *Mycoplasma* spp. For SRLV infection, 25 goats were considered positive by ELISA and/or AGID, and all AGID-positive animals were also positive by ELISA. Concomitant clinical manifestations were observed exclusively in coinfected animals, as shown in Table 2.

**Table 2:** Distribution of clinical manifestations according to infection status for small ruminant lentiviruses (SRLV) and *Mycoplasma agalactiae* in dairy goats

Clinical manifestation	SRLV	M. agalactiae	Coinfected	Negative	Total
Arthritis	0	0	11	0	11
Agalactia	0	1	2	0	3
Mastitis	1	0	2	0	3
Keratoconjunctivitis	1	4	6	0	11
Total animals with clinical signs	2	5	11	0	18
No clinical signs	2	7	10	1	20
Total	4	12	21	1	38

*SRLV* small ruminant lentivirus. Coinfected = animals simultaneously positive for SRLV and *M. agalactiae*. Animals may present more than one clinical manifestation; therefore, the sum of clinical signs exceeds the total number of animals. Values represent the number of affected goats in each infection category

Among goats clinically diagnosed with arthritis (*n* = 11), 54.5% (*n* = 6) had hematocrit values.

below the species-specific reference range. Eight animals (72.7%) presented increased total plasma.

protein concentrations (8.0–9.2 g/dL), and the same proportion showed elevated fibrinogen levels (591–1000 mg/dL). Leukocytosis (14–17.2 × 10³/µL) was observed in approximately 36% of these.

animals (Table 3).

**Table 3:** Hematological parameters of 11 goats clinically diagnosed with arthritis

Parameter	Females										Males		Mean	SD	Reference range
	1	2	3	4	5	6	7	8	9	10		11			
RBC (×10⁶/µL)	14.5	13.8	11.2	10.8	12	11.6	17	9.2	11.4	13.6		10.2	12.3	2.2	8.5–18
Hematocrit (%)	25	23	20	17	20	17	24	15	21	22		23	20.6	3.2	22–38
MCV (fL)	17.3	16.7	17.8	15.7	16.6	14.5	14.1	16.4	18.4	16.2		22.6	16.9	2.3	16–25
TPP (g/dL)	8.4	8.8	6.4	7.6	7.4	7.0	8.4	8.0	9.2	9.0		8.6	8.1	0.9	6–7.5
Fibrinogen (mg/dL)	800	800	200	600	600	300	400	200	600	1000		1000	591	292	100–400
TLC (x10^3^/µL)	14.8	14	9.8	9.5	5.7	11.6	17.2	11.1	18	12.7		15.4	12.7	13.6	4–13
Band neutrophils	0.0	0.0	0.0	0.0	0.0	232	0.0	0.0	0.0	0.0		0.0	21	70	Rare
Seg. neutrophils	7.5	7.0	2.8	4.3	5.3	3.8	2.8	4.0	6.8	7.4		6.9	5.2	1.9	1.2–7.2
Eosinophils	0.0	140	686	95	57	0.0	180	663	0.0	0.0		154	180	253.7	50–650
Basophils	148	0.0	0.0	0.0	0.0	0.0	0.0	0.0	0.0	0.0		0.0	14	44.6	0–120
Lymphocytes	6.2	6.4	6.2	4.8	5.4	7.4	9.8	6.0	10.1	5.2		8.0	6.9	1.8	2–9
Monocytes	888	419	98	380	57	116	516	442	1077	127		308	402.5	329.4	0–550

Legend: *RBC* Red blood cells, *MCV* Mean corpuscular volume, *TPP* Total plasma protein, *TLC* Total leukocyte count, and *SD* standard deviation. Hematological reference ranges according to Jain (1993).

### Laboratory diagnosis and clinical findings

All animals with arthritis were seropositive for SRLV in at least one serological assay. A statistically significant association between arthritis and SRLV seropositivity was observed by indirect ELISA (*P* = 0.01) and when positivity by either AGID or ELISA was considered (*P* = 0.006).

Among synovial fluid samples obtained from animals with arthritis (*n* = 6), all were PCR-positive for *Mollicutes*, and 83.3% were identified as *M. agalactiae*. This agent was also detected in 66.7% (2/3) of ocular swab samples collected from goats with conjunctivitis and in 79.3% (23/29) of the milk samples analyzed. A lower antibody frequency against SRLV was observed in animals with mastitis or agalactia. Milk samples were obtained from four of the six animals presenting these alterations, and *M. agalactiae* was detected in 50% (*n* = 2) of these samples.

Some individual cases demonstrated discordance between clinical and laboratory findings. One animal without clinical signs of arthritis was seropositive for *Mycoplasma* spp. and tested positive for *M. agalactiae* in synovial fluid. Among lactating females without clinical signs of mastitis or agalactia, 84% (21/25) showed simultaneous positivity in milk PCR and serology for *Mycoplasma* spp., whereas two animals were seropositive and PCR-negative; no animals with PCR-positive and seronegative results were observed. Additionally, one goat with conjunctivitis was seronegative for both SRLV and *Mycoplasma* spp. but tested positive for M. agalactiae by PCR in the ocular swab. Another animal with conjunctivitis and agalactia was also seronegative for both agents, although *M. agalactiae* was detected in the milk sample.

Comparison between AGID and indirect ELISA for SRLV diagnosis revealed different positivity rates (31.6% and 65.8%, respectively), with an agreement rate of 65.8% and a Cohen’s kappa coefficient of K = 0.387 (*P* = 0.002) (Table 4).

**Table 4:** Detection frequency of small ruminant lentivirus (SRLV) and *Mycoplasma* spp. by serological and molecular assays according to the presence of arthritis, conjunctivitis, mastitis, and agalactia in dairy goats

Test	Samples	Tested species	Test positivity % (*N*/T)	Arthritis % (*N*/T)	Conjunctivitis % (*N*/T)	Mastitis % (*N*/T)	Agalactia % (*N*/T)
*Small ruminant lentivirus (SRLV)*							
AGID	Serum		31.6 (12/38)	45.4 (5/11)	27.3 (3/11)	33.3 (1/3)	27.3 (2/3)
iELISA			65.8 (25/38)	100 (11/11)	63.6 (7/11)	27.3 (2/3)	27.3 (2/3)
AGID or iELISA			65.8 (25/38)	100 (11/11)	63.6 (7/11)	27.3 (2/3)	27.3 (2/3)
*Mycoplasma* spp.							
iELISA	Serum		34.2 (13/38)	27.3 (3/11)	27.3 (3/11)	27.3 (2/3)	33.3 (1/3)
PCR	Milk	*M. agalactiae*	79.3 (23/29)	54.5 (6/11)	63.6 (7/11)	100 (1/1)	33.3 (1/3)
	Synovial fluid	*Mollicutes*	100 (7/7)	100 (6/6)	100 (3/3)	NP	100 (2/2)
		*M. agalactiae*	85.7 (6/7)	83.3 (5/6)	100 (3/3)	NP	100 (2/2)
		*M. mycoides subsp. capri*	0 (0/7)	0 (0/6)	0 (0/3)	NP	0 (0/2)
	Ocular swab	*Mollicutes*	66.6 (2/3)	50 (1/2)	66.7 (2/3)	NP	NP
		*M. agalactiae*	66.6 (2/3)	50 (1/2)	66.7 (2/3)	NP	NP
		*M. mycoides* subsp. *capri*	0 (0/3)	0 (0/1)	0 (0/2)	NP	NP
		*M. conjunctivae*	0 (0/03)	0 (0/1)	0 (0/2)	NP	NP

*NP* Not performed

**Fig. 4:**
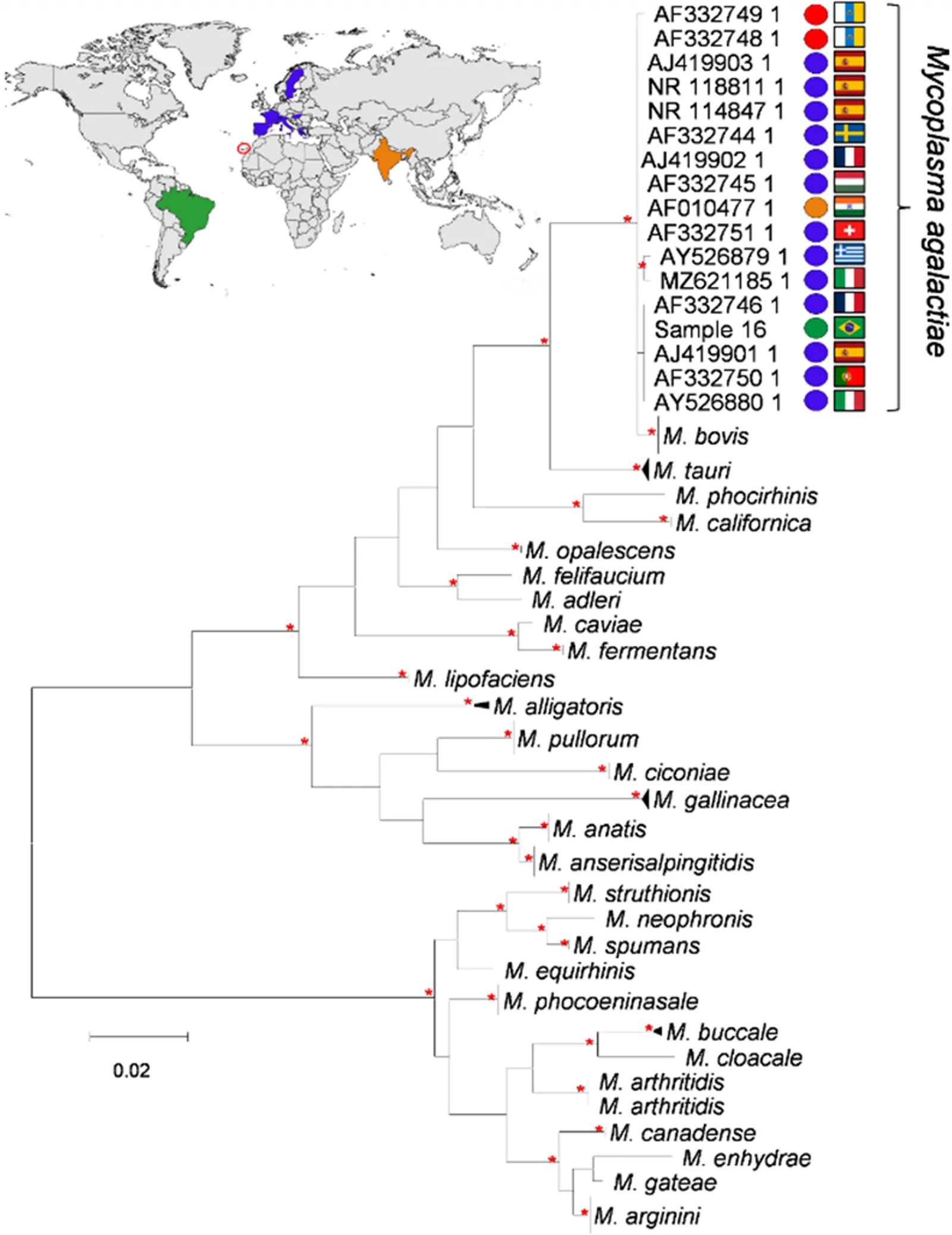
Phylogenetic tree inferred using the Maximum Likelihood method based on 16 S rRNA gene sequences of *Mycoplasma* species. Only bootstrap values ≥ 80% (500 replicates) are shown and indicated by red asterisks at the nodes. *M. agalactiae* sequences are highlighted by geographic origin: Europe (purple), India (orange), the Canary Islands (red), and Brazil (green). GenBank accession numbers are indicated in the taxon labels. The scale bar represents 2% genetic distance (GTR model)

16 S rRNA gene sequence analysis supported the molecular identification of *M. agalactiae* in the clinical samples obtained in this study. The generated sequence clustered within the *M. agalactiae* clade together with reference sequences from different countries, with high bootstrap support, corroborating the species-level identification obtained by PCR and sequencing (Fig. 4).

## Discussion

The present study provides important evidence of coinfection by *Mycoplasma* spp. and SRLV in dairy goats, highlighting the overlap of clinical manifestations observed in the evaluated animals, particularly arthritis, keratoconjunctivitis, and mastitis/agalactia. In addition, the findings emphasize the importance of a multi-sample, multimodal diagnostic approach when investigating infectious diseases in small ruminants, given the diversity of clinical presentations and the potential simultaneous involvement of multiple etiological agents (Damasceno et al. 2020; Migliore et al. 2021; Luján et al. 2025).

Mycoplasmas are considered pantropic organisms in vivo, exhibiting tropism for multiple host tissues, including ocular, mammary, respiratory, reproductive, and articular structures (Razin et al. 1998; Dawood et al. 2022). Consequently, infected animals may develop isolated or combined clinical syndromes, such as keratoconjunctivitis, mastitis/agalactia, pneumonia, reproductive disorders, and arthritis. In small ruminants, mycoplasmosis and lentiviral infection represent important differential diagnoses for one another (Luján et al. 2025). SRLV infections may also cause arthritis, mastitis, and pneumonia. Despite the distinct etiologies, the considerable overlap of clinical signs makes field diagnosis particularly challenging.

Mycoplasmas may be part of the microbiota of different hosts and, under certain circumstances, behave as opportunistic pathogens in ruminants, being reported in both healthy animals and individuals with clinical disease (Franco et al. 2019; Gaeta et al. 2021, 2024). Changes in the host immune status or environmental conditions may favor the clinical expression of these infections. Primary viral infections, for example, can alter the tissue microenvironment and facilitate the proliferation of opportunistic microorganisms (Bosch et al. 2013; Sender et al. 2021).

It is possible that stress associated with transport, herd relocation, changes in management and nutritional management, as well as environmental exposure resulting from high temperatures and the absence of shaded areas on the destination farm, contributed to the exacerbation of physiological stress in the animals. These factors may have favored transient immunosuppression, the clinical expression of pre-existing infections, and dissemination of opportunistic agents within the herd, including SRLV and mycoplasmosis (Razin et al. 1998; Bosch et al. 2013; Migliore et al. 2021).

The detection of antibodies against SRLV in all arthritic animals reinforces the notion that joint involvement is one of the most frequently observed clinical manifestations of lentiviral infection in goats (Luján et al. 2025). Regarding *Mollicutes*, particularly *M. agalactiae*, its presence was confirmed in synovial fluid samples from animals with arthritis, as well as in ocular and milk samples obtained from goats presenting mammary alterations. The detection of *M. agalactiae* in different biological samples is consistent with the systemic behavior described for contagious agalactia and supports the role of *M. agalactiae* in the clinical condition observed in the herd (Jaÿ and Tardy 2019; Migliore et al. 2021). A statistically significant association was observed only between arthritis and SRLV seropositivity, suggesting a potential role of lentiviral infection in the articular manifestations observed in the evaluated goats. Although hematological abnormalities were identified in clinically arthritic goats, some findings, particularly mild anemia, are nonspecific and may also reflect concurrent herd conditions, including chronic inflammatory conditions such as caseous lymphadenitis and papillomatosis, as well as parasitic diseases. This finding highlights the diagnostic complexity of coinfection with mycoplasmas and lentiviruses, as both infections can present similar clinical manifestations, particularly arthritis and mastitis (Migliore et al. 2021; Luján et al. 2025).

Phylogenetic analysis of the 16 S rRNA gene supported the molecular identification of *M. agalactiae* in the clinical samples analyzed. The obtained sequence was grouped within the *M. agalactiae* clade, together with reference sequences retrieved from GenBank, supporting species-level identification (Van Kuppeveld et al. 1992; Chávez González et al. 1995) and reducing the likelihood of misidentification with other *Mycoplasma* species associated with small ruminants, such as *M. conjunctivae* or *M. mycoides* subsp. *capri.*

Another relevant aspect concerns the differences between molecular and serological diagnostic approaches used to investigate mycoplasma infections. PCR allows direct detection of pathogen DNA in clinical samples, whereas serological assays depend on the host antibody response, which may vary with the stage of infection and prior exposure to the agent (Jaÿ and Tardy 2019; Migliore et al. 2021). These differences may partially explain the discordant laboratory findings observed in some animals in the present study, including PCR-positive and seronegative cases.

The higher frequency of SRLV-seropositive animals detected by indirect ELISA compared with AGID further reinforces ELISA’s higher analytical sensitivity. Although AGID shows high specificity and is still widely used in herd screening programs, it has a higher detection threshold, which may result in false-negative results during the early stages of infection or in animals with low antibody titers (Rodrigues et al. 2018). Therefore, the use of more sensitive methods, such as ELISA, either alone or in combination with other serological assays, may contribute to earlier and more accurate detection of SRLV infection in goat herds (Konishi et al. 2011).

Overall, the findings highlight the complexity of *Mycoplasma* spp. and SRLV infections in goats, as well as the broad clinical spectrum associated with coinfections. The results reinforce the need to consider different biological samples and multiple laboratory approaches in the diagnostic investigation of these diseases, particularly in herds presenting multifocal clinical manifestations. The present study does not propose new diagnostic techniques; however, it highlights the usefulness of combining clinical evaluation with serological and molecular methods applied to different biological samples in the investigation of coinfections under field conditions.

It is important to note that the present study was conducted in a single herd, which limits epidemiological extrapolation to broader goat populations. However, the objective was not to estimate population prevalence but rather to characterize, under field conditions, the clinical and diagnostic aspects of coinfection by SRLV and *Mycoplasma* spp. In this context, the findings highlight the diagnostic complexity of these infections when they occur simultaneously and reinforce the need for investigative strategies that combine analysis of multiple biological samples and serological and molecular methods in similar clinical scenarios.

## Conclusion

Coinfection with SRLV and *M. agalactiae* was identified in a dairy goat herd presenting clinical manifestations of arthritis, keratoconjunctivitis, and lactation disorders. The overlap of clinical signs highlights the diagnostic complexity of these diseases under field conditions. The findings reinforce the importance of integrating clinical evaluation with serological and molecular methods, as well as analyzing diverse biological samples, to improve the detection and characterization of infectious diseases in goat herds.

## Supplementary Information

Below is the link to the electronic supplementary material.

GenBank accession numbers, country of origin, species identification, and corresponding references.

Supplementary Material 1

## Data Availability

The data supporting the findings of this study are available from the corresponding author uponreasonable request.
