# Supplementary material for: Concomitant circulation of lentiviruses and Mycoplasma spp. in dairy goats: a clinical and multi-sample diagnostic approach
Source: Vet Res Commun. 2026 Jun 24;50(5):414. doi: 10.1007/s11259-026-11363-5 (PMC13294318; doi:10.1007/s11259-026-11363-5)
Supplement: Supplementary file 1 — Supplementary Material 1 [file 11259_2026_11363_MOESM1_ESM.docx]

Supplementary Table S1. Distribution of isolated and concomitant clinical manifestations according to infection status for small ruminant lentiviruses (SRLV) and *Mycoplasma agalactiae* in dairy goats.

| Clinical manifestation combination | SRLV | *M. agalactiae* | Coinfected | Negative | Total |
| --- | --- | --- | --- | --- | --- |
| Arthritis | 0 | 0 | 3 | 0 | 3 |
| Agalactia | 0 | 1 | 0 | 0 | 1 |
| Mastitis | 1 | 0 | 0 | 0 | 1 |
| Keratoconjunctivitis | 1 | 4 | 0 | 0 | 5 |
| Arthritis + agalactia | 0 | 0 | 1 | 0 | 1 |
| Arthritis + mastitis | 0 | 0 | 1 | 0 | 1 |
| Arthritis + keratoconjunctivitis | 0 | 0 | 4 | 0 | 4 |
| Arthritis + agalactia + keratoconjunctivitis | 0 | 0 | 1 | 0 | 1 |
| Arthritis + mastitis + keratoconjunctivitis | 0 | 0 | 1 | 0 | 1 |
| Total animals with clinical signs | 2 | 5 | 11 | 0 | 18 |
| No clinical signs | 2 | 7 | 10 | 1 | 20 |
| Total | 4 | 12 | 21 | 1 | 38 |

SRLV = small ruminant lentiviruses. Coinfected = animals simultaneously positive for SRLV and *M. agalactiae*.
